# The relationship between immune status as measured by stimulated ex-vivo tumour necrosis factor alpha levels and the acquisition of nosocomial infections in critically ill mechanically ventilated patients

**DOI:** 10.1186/s40635-020-00344-w

**Published:** 2020-09-16

**Authors:** Gabrielle Levin, J. Gordon Boyd, Andrew Day, Miranda Hunt, David M. Maslove, Patrick Norman, Nicole O’Callaghan, Stephanie Sibley, John Muscedere

**Affiliations:** 1 Kingston Health Sciences Center, Kingston, Ontario Canada; 2 Department of Critical Care Medicine, Queen’s University, Watkins C, 76 Stuart Street, Kingston, Ontario K7L 2V3 Canada

**Keywords:** Immune dysfunction, critically ill, TNF- α, nosocomial infections

## Abstract

**Introduction:**

Immunological dysfunction is common in critically ill patients but its clinical significance and the optimal method to measure it are unknown. The level of tumor necrosis factor alpha (TNF-α) after ex-vivo whole blood stimulation with lipopolysaccharide (LPS) has been proposed as a possible method to quantify immunological function. We hypothesized that in a cohort of critically ill patients, those with a lower post-stimulation TNF-α level would have increased rates of nosocomial infections (NIs) and worse clinical outcomes.

**Methods:**

A secondary analysis of a phase 2 randomized, multi-centre, double-blinded placebo-controlled trial. As there was no difference between treatment and control arms in outcomes and NI rate, all the patients were analyzed as one cohort. On enrolment, day 4, 7, and weekly until day 28, whole blood was incubated with LPS ex-vivo and subsequent TNF-α level was measured. Patients were grouped in tertiles according to delta and peak TNF-α level. The primary outcome was the association between NIs and tertiles of TNF-α level post LPS stimulation; secondary outcomes included ICU and 90-day mortality, and ICU and hospital length of stay.

**Results:**

Data was available for 201 patients. Neither the post LPS stimulation delta TNF-α group nor the peak TNF-α post-stimulation group were associated with the development of NIs or clinical outcomes. Patients in the highest tertile for post LPS stimulation delta TNF-α compared to the lowest tertile were younger [61.1 years ± 15.7 vs. 68.6 years ± 12.8 standard deviations (SD) in the lowest tertile], had lower acuity of illness (APACHE II 25.0 ± 9.7 vs. 26.7 ± 6.1) and had lower baseline TNF-α (9.9 pg/mL ± 19.0 vs. 31.0 pg/mL ± 68.5). When grouped according to peak post-stimulation TNF-α levels, patients in the highest tertile had higher serum TNF-α at baseline (21.3 pg/mL ± 66.7 compared to 6.5 pg/mL ± 9.0 in the lowest tertile).

**Conclusion:**

In this prospective multicenter study, ex-vivo stimulated TNF-α level was not associated with the occurrence of NIs or clinical outcomes. Further study is required to better ascertain whether TNF levels and ex-vivo stimulation can be used to characterize immune function in critical illness and if other assays might be better suited to this task.

## Introduction

Nosocomial infections (NI), also known as healthcare-associated infections (HAI) complicate 30% of intensive care unit (ICU) admissions and are associated with increased mortality and morbidity including both longer ICU and hospital stays [1, 2]. In the ICU, mechanically ventilated patients are at the highest risk for NIs, likely due to the presence of multiple risk factors [3]. An increasingly recognized potential risk factor for infection in the critically ill is immunological dysfunction, which has been described in both critically ill adults and children since the 1980s [4, 5]. Immune dysfunction can occur in patients who experience severe trauma, are post-operative or in patients with sepsis [4, 6, 7]. The exact mechanism of immune dysfunction is unknown, but is likely related to abnormal regulation of inflammation, as well as the development of tolerance to stimulation by the immune system.

Studies that have examined immune dysfunction and the relationship with patient outcomes have reported inconsistent findings. In patients with either sepsis or trauma, most studies show a significantly lower survival rate in patients with prolonged low human leukocyte antigen (HLA)-DR expression by monocytes, as well as higher rates of major infections compared to those patients who only had a transient or less severe drop in HLA-DR expression [8–13]. However, Perry and colleagues did not find a relationship between HLA-DR expression in septic patients and patient outcomes [14]. Further studies also found that HLA-DR expression had poor discriminating power in identifying septic patients at high risk of dying [15]. Oberholzer et al. found that selected baseline cytokines including interleukin (IL)-6 and soluble tumor necrosis factor were helpful in predicting patient outcomes, while other cytokines, including TNF-α, as well as the change in cytokine concentrations over time, were not predictive of patient outcomes [16].

The relationship between immune dysfunction and the acquisition of NIs remains uncertain. Studies looking at cytokine levels in patients admitted with sepsis and the correlation with the development of NIs, have yielded mixed results [17, 18]. One study found no difference in cytokine levels between patients admitted with sepsis who later developed NIs, and those admitted with sepsis who did not develop NIs [17]. However, Van Vught et al found significant elevations of inflammatory cytokines in patients with sepsis who developed NIs as compared to patients who did not, and suggested that these patients have concomitant hyper-inflammation and immune suppression to a greater degree than those patients who only had sepsis [18]. Immune dysfunction has also been studied in the development of NIs after trauma and elective surgery [5, 6, 19–21]. These studies suggested that distinct inflammatory marker patterns exist in patients who develop NIs. Currently, there are few studies that look at cytokine levels as they relate to NIs in all patients admitted to an ICU. Further, it is still unknown whether higher or lower levels of pro-inflammatory cytokines correlate to the development of NIs, and whether admission cytokine levels can help predict who develops these infections.

The best way to measure immune dysfunction is unknown, but some studies suggest that TNF-α levels in lipopolysaccharide (LPS)-stimulated whole blood are more accurate in predicting patient outcomes than using HLA-DR expression [22, 23]. LPS, also known as endotoxin, is a component of the outer membrane of gram-negative bacteria and is known to stimulate monocytes to release cytokines, including TNF-α. Studies of TNF-α levels post-LPS stimulation in healthy adults, show significant variation in both baseline levels of TNF-α and levels post-LPS stimulation [24–28]. Patients with middle range initial levels of TNF-α had a response to LPS stimulation, while people with high levels and some with low levels of TNF-α did not respond [28]. Bruunsgard et al and von Haehling et al showed that there are differences in immune stimulation between age groups, but their results are conflicting [29]. There is little research on the use of an *ex-vivo* LPS assay in critically ill patients, but the data available suggests that there is less of a response to LPS in patients in the ICU versus healthy patients [22, 30]. Further, there is minimal data on whether TNF-α response to LPS is related to patient outcomes. Ploder et al. and Heagy et al. suggested that patients who had a lower TNF-α response to LPS at baseline had a worse prognosis than patients who had a higher TNF-α response [22, 31]. Few studies have looked at TNF-α response to LPS as it evolves over the course of a patient’s admission to the ICU.

We hypothesized that patients with lower peak levels and smaller changes (delta) of post-stimulation TNF-α on ICU admission would develop more NIs, have longer ICU and hospital lengths of stays, and increased mortality. To test our hypothesis, we conducted a descriptive analysis to describe the characteristics of an ex-vivo whole blood LPS stimulation assay in critically ill, mechanically ventilated patients in the ICU as measured by change in the level of TNF-α, and explore how levels of TNF-α after stimulation by an LPS assay are associated with clinical outcomes including mortality and the development of NIs.

## Methods

### Design

A secondary analysis of the PREVAIL study, a phase 2 randomized, multi-centre, double-blinded placebo controlled trial conducted in five Canadian tertiary ICUs studying the effect of lactoferrin on the acquisition of NIs [32]. The protocol for this study has been published and the trial was registered at www.clinicaltrials.gov on 18 November 2013 (registration number NCT01996579) [33].

### Patients

Adult patients (≥ 18 years old) receiving invasive mechanical ventilation on ICU admission and who were expected to receive mechanical ventilation for > 72 hours were included in the original trial. Patients who met the following criteria were excluded:

- who were expected to be in the ICU for < 72 hours,
- immunocompromised patients including those post-organ transplant, patients with Acquired Immunodeficiency Syndrome (AIDS), neutropenia, use of glucocorticoids (> 20 mg/day of prednisone equivalent for more than 6 months), use of immunosuppressant medication (e.g. patients with rheumatological conditions on methotrexate, etc.)
- patients with end stage liver disease or fulminant liver failure
- pregnant or lactating patients
- patients with a life expectancy of less than six months due to pre-existing conditions
- enrollment in other interventional trials.

All patients were followed for the duration of their ICU or until day 28 for the acquisition of NIs. During the study, if there was a prescription of a new antibiotic or the patient was investigated for infection with the collection of microbial cultures, a suspicion of infection event was triggered; the attending physician was then asked to assess the probability of infection; definite, probable and possible which was then centrally adjudicated to ensure consistency [33]. The definitions used for each category of NI are outlined further in the supplemental digital content (Supplementary Digital Content 1: Definitions of nosocomial infections). These suspicions were then adjudicated by an assessor blinded to treatment allocation for the presence of infection. Discrepancies between the attending physician and adjudicator were resolved by consensus.

As part of the original protocol, laboratory investigations included measurement of the following cytokines on admission to the ICU, as well as on days 4 and 7, and then weekly until 28 days post-admission: IL-6, IL-10, and TNF-α. Immune function was measured over time by the levels of TNF-α in response to an ex-vivo LPS stimulation assay on those same days. The LPS stimulation assay was conducted by sampling the patient’s blood in sodium heparin tubes. Fifty (50) μl of whole blood was then pipetted into 500 μl of LPS stimulation solution, at a concentration of 500 pg/mL [34]. The time between collection of the blood and processing was less than 30 minutes. The samples were incubated at 37°C for 4 hours and then centrifuged. The supernatant was pipetted into the microcentrifuge tubes, stored at -80°C and then analyzed.

The primary outcome for this study was the occurrence of NIs acquired during the ICU admission in relation to the tertiles of delta and peak TNF-α levels post-LPS stimulation. NIs were defined as an infection occurring after 72 hours of ICU admission. For the purposes of this analysis we considered all categories of suspected infection including possible, probable and definite as positive [35]. Secondary outcomes included clinical outcomes and laboratory outcomes. Clinical outcomes included ICU and hospital length of stay (LOS) and ICU, hospital and 90-day mortality. Severity of illness was measured using the APACHE II and Sequential Organ Failure Assessment (SOFA) scores. The SOFA score was recorded throughout the ICU admission.

### Statistical Analysis

#### Sample Size

The sample size was based on the clinical study [33]. A total of 214 patients were enrolled; blood samples for analysis were available for analysis in 201. The PREVAIL study found no difference in outcomes based on allocation so both allocation groups were combined for this secondary analysis.

#### Statistical Methods

The primary variable of interest (TNF-α response) was represented in two ways: the maximum TNF-α level post-LPS challenge (peak TNF-α) and the change from pre-challenge to post-challenge levels (delta TNF-α). As done in prior studies by Heagy, W., et al. and Mózes, T et al, patients were divided into tertiles based on the level of TNF-α in response to ex-vivo LPS stimulation assay on admission to the ICU [31, 36]. Any patients without a peak TNF-α measure were excluded from both baseline analyses, while patients without a pre-challenge TNF-α measure reported were excluded from the delta TNF-α analysis.

Clinical outcomes represented as continuous variables, including the total number of adjudicated nosocomial infections and the rate of adjudicated nosocomial infections per patient, were compared between TNF-α tertiles using the Kruskal-Wallis test. Categorical and binary variables, including mortality and source of adjudicated infection, were compared using the Chi-Squared test. This comparison method was mirrored for all other variables that were compared, with one exception: primary diagnosis on admission was compared using Fisher’s exact test due to small cell sizes. Because the large number of diagnosis categories made this computationally prohibitive, a Monte Carlo simulation was employed.

Multivariable logistic regression models for each of peak TNF-α and delta TNF-α data were created using the occurrence of ever having a nosocomial infection during the hospital stay as the dependent variable (See Supplemental Content 2: Multivariable analysis). Age, sex, medical or surgical admission type, lactoferrin versus placebo arm, APACHE II score, and severe sepsis were included as covariates, in addition to either the peak or delta TNF-α tertile (using the highest tertile as the referent).

#### Multiple Time Point Analysis

Data on TNF-α response levels were collected on ICU days 0, 4, 7, 14, 21 and 28. Box plots of the distributions of peak TNF-α, delta TNF-α and baseline serum TNF-α were created. A panel of profile plots showing the change from baseline to peak TNF-α for each patient, on each day, was produced. A longitudinal plot showing the average baseline and peak TNF-α values on each day was also produced, as was a similar longitudinal plot of the change in TNF-α values for each patient on each day.

## Results

**Fig. 1:**
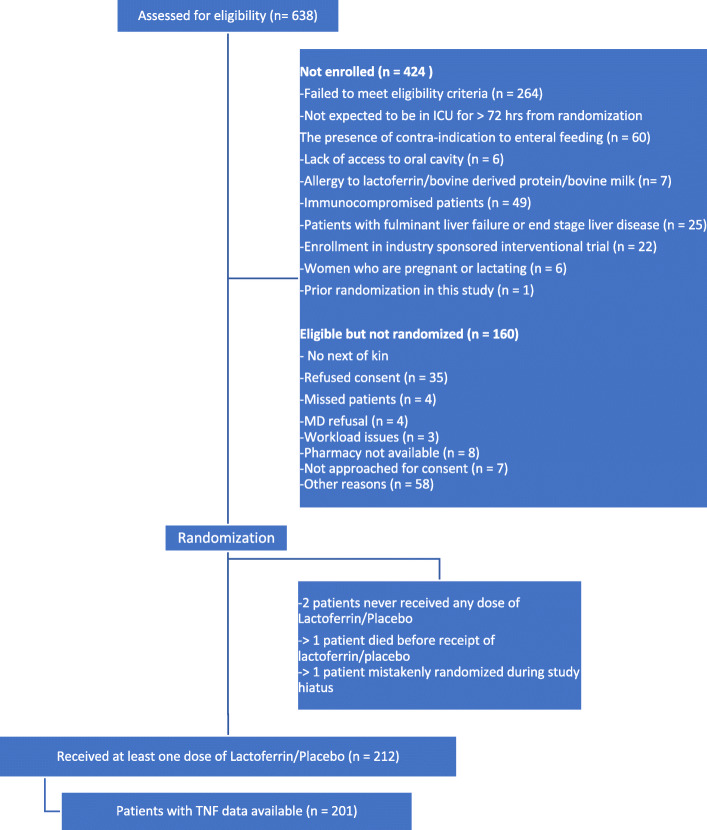
Study Consort Diagram

Data for the peak and delta TNF-α analyses were available for 201 patients and 200 patients, respectively (Figure 1). No significant differences were found between delta TNF-α tertiles or peak TNF-α tertiles for any measure of NIs. Tables 1 and 2 report the results of comparing clinical outcomes including suspected and adjudicated infections, mortality, and length-of-stay measures between TNF-α response tertiles. No significant differences were found between delta TNF-α tertiles or peak TNF-α tertiles for any measure of NIs.

**Table 1:** Outcomes grouped by peak TNF-α post-LPS challenge

	Tertile 1 (Low TNF-α levels) (***n*** = 67)	Tertile 2 (Medium TNF-α levels) (***n***=67)	Tertile 3 (High TNF-α levels) (***n***=67)	Total cohort (***n***=201)	***P***-value
**Suspected nosocomial infection:** N (%)	31 (46%)	39 (58%)	39 (58%)	109 (54%)	
**Total positive adjudicated infections:** n	16	22	29	67	0.494
**Adjudicated positive infections per subject:** Mean ± SD	0.2 ± 0.5	0.3 ± 0.7	0.4 ± 0.8	0.3 ± 0.7	0.066
**Source of positive infections**					
Surgical	2 (12.5%)	1 (4.5%)	1 (3.4%)	4 (2.0%)	
Skin-soft tissue	0 (0.0%)	1 (4.5%)	2 (6.9%)	3 (1.5%)	
Catheter BSI	0 (0.0%)	1 (4.5%)	4 (13.8%)	5 (2.5%)	
Primary BSI	0 (0.0%)	3 (13.6%)	3 (10.3%)	6 (3.0%)	
UTI	0 (0.0%)	4 (18.2%)	6 (20.7%)	10 (14.9%)	
Intra-abdominal	0 (0.0%)	2 (9.1%)	0 (0.0%)	2 (1.0%)	
Lower RTI	6 (37.5%)	2 (9.1%)	2 (6.9%)	10 (5.0%)	
ICU Pneumonia	6 (37.5%)	7 (31.8%)	11 (37.9%)	24 (11.9%)	
Other	2 (12.5%)	1 (4.5%)	0 (0.0%)	3 (1.5%)	
**ICU Mortality:** n (%)	21 (31.3%)	21 (31.3%)	23 (34.3%)	65 (32.3%)	0.913
**Hospital mortality:** n (%)	23 (34.3%)	21 (31.3%)	26 (38.8%)	70 (34.8%)	0.659
**90-day mortality:** n (%)	24 (35.8%)	24 (35.8%)	28 (41.8%)	76 (37.8%)	0.713
**ICU length of stay**					0.552
Median [IQR]	9.1 [5.5 to 13.8]	9.0 [6.9 to 13.3]	9.0 [5.3 to 15.9]	9.0 [5.9 to 15.2]	
**Hospital length of stay**					0.237
Median [IQR]	17.6 [7.7 to 30.3]	14.6 [8.5 to 33.9]	19.1 [9.0 to 34.0]	16.8 [8.5 to 32.3]

**Table 2:** Outcomes grouped by change in TNF-α post-LPS challenge

	Tertile 1 ((Post-LPS) - (Pre-LPS) <= -3.09 (***n***=66)	Tertile 2 ((Post-LPS) - (Pre-LPS) <= 19.42 (***n***=67)	Tertile 3 ((Post-LPS) - (Pre-LPS) > 19.42 (***n***=67)	Total cohort (***n***=200)	***P***-value
**Suspected nosocomial infection:** N (%)	34 (52%)	35 (52%)	39 (58%)	108 (54%)	
**Total positive adjudicated infections:** n	21	16	29	66	0.489
**Adjudicated positive infections per subject:** Mean ± SD	0.3 ± 0.7	0.2 ± 0.5	0.4 ± 0.8	0.3 ± 0.7	0.22
**Source of positive infections**					
Surgical	2 (9.5%)	1 (6.3%)	1 (3.4%)	4 (2.0%)	
Skin-soft tissue	1 (4.8%)	0 (0.0%)	2 (6.9%)	3 (1.5%)	
Catheter BSI	1 (4.8%)	0 (0.0%)	4 (13.8%)	5 (2.5%)	
Primary BSI	3 (14.3%)	0 (0.0%)	3 (10.3%)	6 (3.0%)	
UTI	2 (9.5%)	2 (12.5%)	6 (20.7%)	10 (14.9%)	
Intra-abdominal	1 (4.8%)	1 (6.3%)	0 (0.0%)	2 (1.0%)	
Lower RTI	2 (9.5%)	6 (37.5%)	2 (6.9%)	10 (5.0%)	
ICU Pneumonia	9 (42.9%)	4 (25.0%)	10 (34.5%)	23 (11.5%)	
Other	0 (0.0%)	2 (12.5%)	1 (3.4%)	3 (1.5%)	
**ICU Mortality:** n (%)	24 (36.4%)	20 (29.9%)	21 (31.3%)	65 (32.5%)	0.703
**Hospital mortality:** n (%)	25 (37.9%)	21 (31.3%)	24 (35.8%)	70 (35.0%)	0.721
**90-day mortality:** n (%)	26 (39.4%)	24 (35.8%)	26 (38.8%)	76 (38.0%)	0.901
**ICU length of stay**					0.743
Median [IQR]	9.1 [5.5 to 13.8]	9.0 [6.9 to 13.3]	9.0 [5.3 to 15.9]	9.0 [5.9 to 15.2]	
**Hospital length of stay**					0.699
Median [IQR]	17.6 [7.7 to 30.3]	14.6 [8.5 to 33.9]	19.1 [9.0 to 34.0]	16.8 [8.5 to 32.3]	

The characteristics of patients when grouped according to peak TNF- α tertiles revealed statistically significant differences in patient characteristics in primary diagnosis, baseline serum TNF-α levels, peak TNF-α levels and delta TNF-α levels (Table 3). Patients in the highest tertile had the highest baseline TNF-α levels [21.3 pg/mL ± 66.7 standard deviations (SD) compared to 6.5 pg/mL ± 9.0 in the lowest TNF-α tertile], the highest peak TNF-α levels [255.4 pg/mL ± 299.4 versus 0.6 pg/mL ± 0.5 in the lowest TNF-α tertile], and the greatest change in TNF-α levels [234.1 pg/mL ± 313.9 compared to -6.0 pg/mL ± 9.0 in the lowest TNF-α tertile]. There was no statistically significant difference between age, sex, APACHE II score, or requirement of vasopressor/inotrope support.

**Table 3:** Baseline characteristics grouped by peak TNF-α levels post-LPS challenge

	Tertile 1 (Low TNF-α Levels) (***n***=67)	Tertile 2 (Medium TNF-α Levels) (***n***=67)	Tertile 3 (High TNF-α Levels) (***n***=67)	Total Cohort (***n***=201)	***P***-value
**Age:** Mean ± SD (range)	65.9 ± 15.4	64.8 ± 13.9	61.4 ± 15.8	64.0 ± 15.1)	0.215
**Sex:** Female	36 (53.7%)	34 (50.7%)	27 (40.3%)	97 (48.3%)	0.263
**Primary Diagnosis**					0.001
Cardiovascular	10 (14.9%)	15 (22.4%)	7 (10.4%)	32 (15.9%)	
Respiratory	27 (40.3%)	14 (20.9%)	15 (22.4%)	56 (27.9%)	
Neurologic	12 (17.9%)	3 (4.5%)	15 (22.4%)	30 (14.9%)	
Sepsis	10 (14.9%)	20 (29.9%)	14 (20.9%)	44 (21.9%)	
Trauma	5 (7.5%)	3 (4.5%)	7 (10.4%)	15 (7.5%)	
Other	3 (4.5%)	12 (17.9%)	9 (13.4%)	24 (11.9%)	
**APACHE II**: Mean ± SD	24.2 ± 7.0	25.6 ± 7.8	25.5 ± 9.5	25.1 ± 8.1	0.646
**Vasopressor support:** N (%)	52 (77.6%)	54 (80.6%)	44 (65.7%)	150 (74.6%)	0.11
**Temperature:** Mean **°**C ± SD	37.9 ± 0.9	37.6 ± 0.8	38.0 ± 1.0	37.9 ± 0.9	0.08
**On Antibiotics:** N (%)	43 (64.2%)	42 (62.7%)	41 (61.2%)	126 (62.7%)	0.938
**Positive culture 48 hrs prior to or after randomization:** N (%)	59 (88.1%)	60 (89.6%)	53 (79.1%)	172 (85.6%)	0.177
**Adjudicated positive culture 48 hours prior to or after randomization:** N (%)	43 (64.2%)	46 (68.7%)	40 (59.7%)	129 (64.2%)	0.557
**Highest white blood cell:** mean ± SD	14.5 ± 6.0	17.3 ± 9.4	17.0 ± 8.1	16.3 ± 8.0	0.277
**Serum TNF-α:** Mean pg/mL ± SD	6.5 ± 9.0	16.4 ± 27.7	21.3 ± 66.7	14.8 ± 42.4	**<0.001**
**Post-LPS TNF-α:** Mean pg/mL ± SD	0.6 ± 0.5	9.3 ± 8.0	255.4 ± 299.4	88.4 ± 208.9	**<0.001**
**Change in TNF-α:** pg/mL	-6.0 ± 9.0	-7.0 ± 29.1	234.1 ± 313.9	74.1 ± 214.4	**<0.001**
**Serum IL-6:** Mean pg/mL ± SD	483.2 ± 2823.8	1229.5 ± 5551.9	197.0 ± 485.6	636.6 ± 3615.2	0.081
**Serum IL-10:** Mean pg/mL ± SD	49.6 ± 81.6	143.2 ± 690.2	51.1 ±74.2	81.3 ± 403.9	0.700

When patients were grouped according to delta TNF-α tertiles, statistically significant differences in patient characteristics were found in age, primary diagnosis, requirement of vasopressor/inotrope support, APACHE II score, baseline serum TNF-α levels, and peak TNF-α levels (Table 4). Patients in the highest tertile compared to the lowest tertile were younger [61.1 years ± 15.7 vs. 68.6 years ± 12.8 SD in the lowest tertile], had lower acuity of illness [APACHE II 25.0 ± 9.7 vs. 26.7 ± 6.1], lower baseline TNF-α levels [9.9 pg/mL ± 19.0 vs. 31.0 pg/mL ± 68.5] and had the highest post-LPS TNF-α level [253.0 pg/mL ± 301.0 vs. 6.4 pg/mL ± 18.0 in the lowest tertile]. No statistically significant differences were found between sex, and serum IL-10 levels.

**Table 4:** Baseline characteristics grouped by change in TNF-α post-LPS challenge

	Tertile 1 ((Post-LPS) - (Pre-LPS) <= -3.09 (***n***=66)	Tertile 2 ((Post-LPS) - (Pre-LPS) <= 19.42 (***n***=67)	Tertile 3 ((Post-LPS) - (Pre-LPS) > 19.42 (***n***=67)	Total Cohort (***n***=200)	***P***-value
**Age:** Mean ± SD	68.6 ± 12.8	62.6 ± 15.7	61.1 ± 15.7	64.1 ± 15.1	**0.021**
**Sex:** Female	36 (54.5%)	32 (47.8%)	29 (43.3%)	97 (48.5%)	0.425
**Primary Diagnosis**					**0.045**
Cardiovascular	13 (19.7%)	12 (17.9%)	7 (10.4%)	32 (16.0%)	
Respiratory	19 (28.8%)	21 (31.3%)	16 (23.9%)	56 (28.0%)	
Neurologic	5 (7.6%)	9 (13.4%)	15 (22.4%)	29 (14.5%)	
Sepsis	13 (19.7%)	17 (25.4%)	14 (20.9%)	44 (22.0%)	
Trauma	3 (4.5%)	6 (9.0%)	6 (9.0%)	15 (7.5%)	
Other	13 (19.7%)	2 (3.0%)	9 (13.4%)	14 (7.0%)	
**APACHE II:** Mean ± SD	26.7 ± 6.1	23.8 ± 7.7	25.0 ± 9.7	25.2 ± 8.0	**0.037**
**Vasopressor support:** N (%)	59 (89.4%)	49 (73.1%)	42 (62.7%)	150 (75.0%)	**0.002**
**Temperature:** Mean **°**C ± SD	37.9 ± 0.8	37.8 ± 1.0	38.0 ± 0.9	37.9 ± 0.9	0.397
**On Antibiotics**					0.388
N (%)	46 (69.7%)	40 (59.7%)	40 (59.7%)	126 (63.0%)	
**Positive culture 48 hrs prior to/after randomization:** N (%)	58 (87.9%)	60 (89.6%)	54 (80.6%)	172 (86.0%)	0.284
**Highest white blood cell:** mean ± SD	15.5 ± 8.0	17.0 ± 8.2	16.4 ± 8.0	16.3 ± 8.0	0.617
**Serum TNF-α:** Mean pg/mL ± SD	31.0 ± 68.5	3.7 ± 3.4	9.9 ± 19.0	14.8 ± 42.4	**<0.001**
**Post-LPS TNF-α:** Mean pg/mL ± SD	6.4 ± 18.0	6.0 ± 7.8	253.0 ± 301.0	88.9 ± 209.3	**<0.001**
**Serum IL-6:** Mean pg/mL ± SD	1600.6 ± 6195.2	130.5 ± 443.0	200.9 ± 484.8	636.6 ± 3615.2	0.01
**Serum IL-10:** Mean pg/mL ± SD	147.2 ± 695.7	45.6 ± 75.3	52.7 ± 74.8	81.3 ± 403.9	0.775

No significant differences were observed in any mortality or length of stay measure for either delta TNF-α tertiles or peak TNF-α tertiles. In the multivariate logistic regression models, neither the delta TNF-α or peak TNF-α were associated with the development of NIs when controlling for the co-variates of severity of illness as measured by APACHE II score, admission diagnosis, sex, age or lactoferrin or placebo arm (Supplementary Digital Content 2: Multivariate analysis).

### Multiple Time Point Analysis

**Fig. 2:**
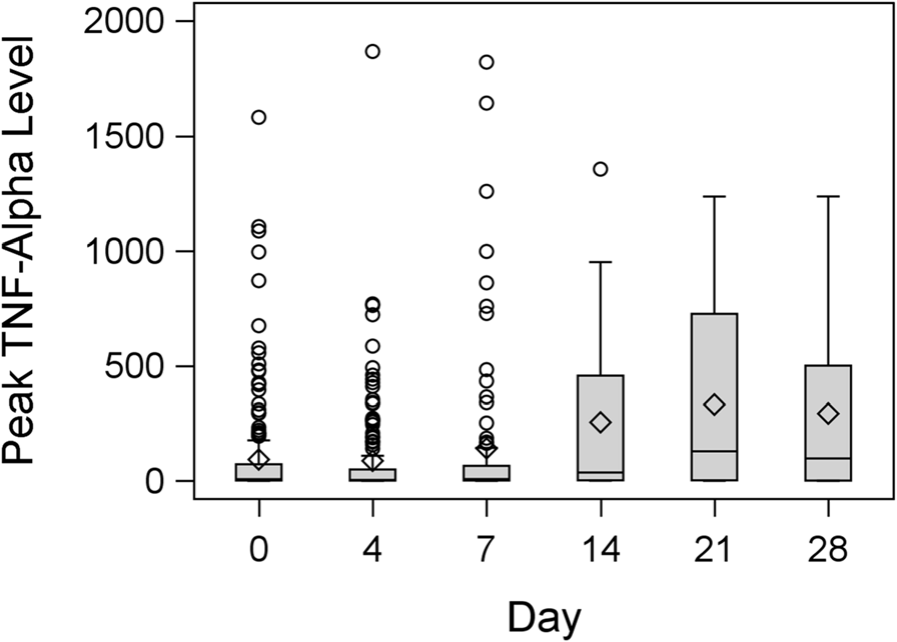
Box plot showing distribution of post-challenge peak TNF-α level on ICU stay day number 0, 4, 7, 14, 21, 28

**Fig. 3:**
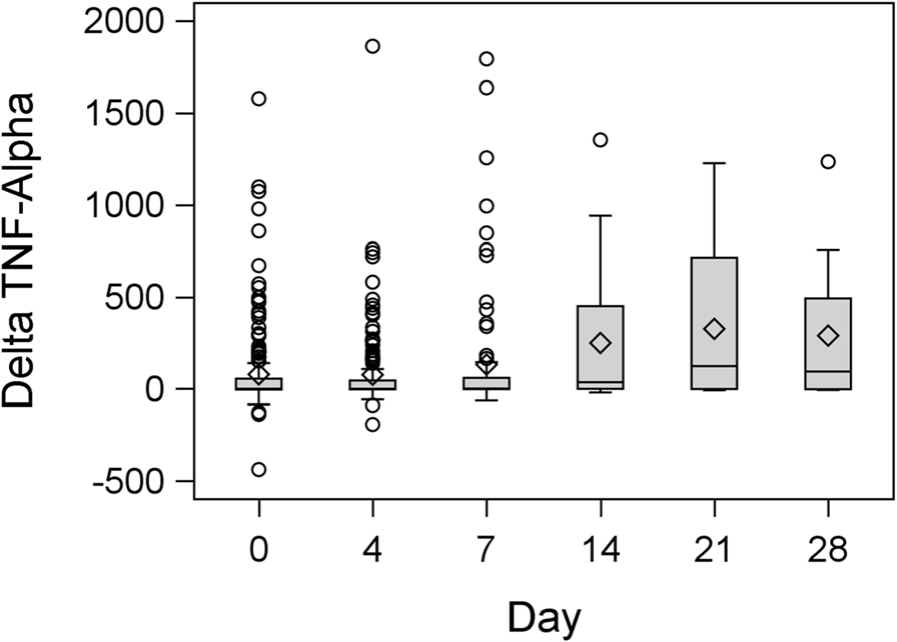
Box plot showing distribution of post-challenge delta TNF-α level on ICU stay day number 0, 4, 7, 14, 21, 28

**Fig. 4:**
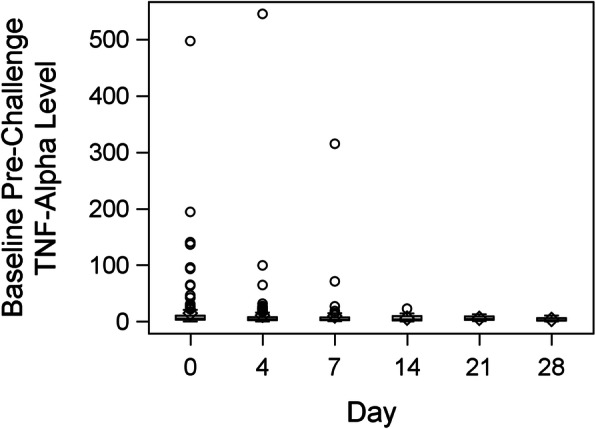
Box plot showing distribution of baseline pre-challenge TNF-α level on ICU stay day number 0, 4, 7, 14, 21, 28

Box plots of the distributions of peak TNF-α, delta TNF-α and baseline serum TNF-α are presented in Figures 2, 3 and 4, respectively. Peak TNF-α levels and delta TNF-α tended to be relatively low on days 0, 4 and 7, with a slight increase on days 14, 21 and 28. Baseline pre-challenge TNF-α levels remained low on all days, with no important day-to-day changes appearing. These results were corroborated by the panel of profile plots and the average change appeared fairly stagnant on days 0, 4, and 7 but increased on days 14, 21 and 28.

## Discussion

In this analysis of the PREVAIL dataset, we found that there was a wide range of TNF- α response to ex-vivo LPS stimulation. While most patients showed very little TNF- α response, a relatively small subset of patients exhibited a marked increase in TNF- α levels following LPS stimulation. There were baseline differences in clinical characteristics across TNF- α tertiles (both peak and delta), but these did not carry through to the outcomes. Neither admission *ex-vivo* stimulated peak TNF-α level, nor the amount of change in TNF-α level post-stimulation were associated with the occurrence of NIs or clinical outcomes.

Immune dysfunction is common in critically ill patients, yet the best method to evaluate the immune system in this setting is unknown. Measuring TNF-α levels post-stimulation by an LPS assay has been suggested as a way to quantify function of the immune system, but previously reported associations between stimulated TNF-α levels and patient outcomes have been inconsistent. In our study, the patients in the lowest peak TNF-α tertile had lower initial TNF-α levels and a smaller overall change in their TNF-α levels, suggesting that their immune system did not respond appropriately to stimulation by LPS. Unlike other studies however, the patients in our study who had a lower immune response did not have worse outcomes than patients who had a higher overall TNF-α response [8, 22, 31].

Previous studies have examined immunosuppression over time in critically ill patients and suggested that it is associated with worse outcomes, but they have not looked specifically at stimulated TNF-α levels [8–13]. Our multiple time point analysis did not find any difference in outcome between patients with prolonged lower TNF-α levels and those with only briefly suppressed levels. Ours is not the only study to find no relationship between immune dysfunction and outcomes, although other studies have looked at HLA-DR expression in critically ill patients rather than TNF-α [14, 15].

As with other studies that have looked at immune dysfunction in critically ill patients, it is difficult to consider all patients admitted to the ICU as a whole, given that patients admitted with sepsis are presumably different than those who are admitted with a primarily neurological condition, to mention one of many possible distinctions. This may be part of the reason there is such a large range in the TNF-α levels. Further, it may be part of the reason we did not find a correlation between TNF-α levels and outcomes, as other studies have examined solely patients with trauma or sepsis [22].

Our study has a number of strengths, including its multicenter design, a relatively large sample size, the availability of reliable infection status based on expert adjudication, and the longitudinal profiling of immune function over the course of the ICU stay. There are, however, some limitations. First, this study was a post-hoc analysis of a randomized controlled trial, meant to be hypothesis generating and was not specifically powered for the primary of outcome differences in nosocomial infections between tertiles of TNF-α response. Second, we included all critically ill patients undergoing invasive mechanical ventilation, regardless of their primary diagnosis. Non-mechanically ventilated patients were excluded since it is thought that endotracheal intubation increases the risk for the development of NIs, and thus these patients would be more likely to benefit from a strategy to prevent NIs [3, 33]. Examining all critically ill patients regardless of ventilation status may be helpful, as patients with other indwelling devices (e.g. central venous catheters, hemodialysis lines) are also at risk of developing NIs and may have immune dysfunction [37]. Third, infection prevention methods were not protocolized in our study. This may have affected the rates of NIs, but is likely more representative of real practice. Fourth, the decision to analyze patients by tertiles was somewhat arbitrary, but there is precedent in other studies that have used either tertiles or quartiles [31, 36]. There is no standardized way of analyzing this data at this time, and it is possible that other strategies for the statistical analysis could have yielded different results.

## Conclusion

The measurement of immunological function in critically ill patients and the correlation with patient centered outcomes is an unmet need. Within the limitations of this study, we found that TNF-α levels in LPS stimulated *ex-vivo* blood are not associated with clinical outcomes. Further study is required to evaluate the ability of this assay to quantify immune function over the course of critical illness, the optimal method for measuring and analyzing the longitudinal TNF- α response to LPS stimulation, and the utility of other biomarkers for characterizing immune dysfunction in critical illness.

## Supplementary information

Additional file 1: Supplementary Digital Content 1: Definitions of nosocomial infections. Supplementary Digital Content 2: Multivariate analysis.

## Data Availability

The datasets used and during the current study are available from the corresponding author on reasonable request.

## Abbreviations

AIDS: Acquired Immunodeficiency Syndrome
HAI: Healthcare-associated infections
HLA: Human leukocyte antigen
ICU: Intensive care unit
IL: Interleukin
LPS: Lipopolysaccharide
NI: Nosocomial infection
SD: Standard deviation
SOFA: Sequential Organ Failure Assessment
TNF-α: Tumor necrosis factor alpha
