# Supplementary material for: The relationship between immune status as measured by stimulated ex-vivo tumour necrosis factor alpha levels and the acquisition of nosocomial infections in critically ill mechanically ventilated patients
Source: Intensive Care Med Exp. 2020 Sep 16;8:55. doi: 10.1186/s40635-020-00344-w (PMC7494693; doi:10.1186/s40635-020-00344-w)
Supplement: Supplementary file 1 — Supplementary Digital Content 1: Definitions of nosocomial infections. Supplementary Digital Content 2: Multivariate analysis. [file 40635_2020_344_MOESM1_ESM.docx]

**Supplementary Material**

**Supplementary Digital Content 1: Definitions of nosocomial infections**

Categories of infection were modified from the definitions developed by the International Sepsis Forum Consensus Conference^[[1]](#footnote-1)^. Given the uncertainty around the diagnosis of infection, each type of infection has been rated: “**Definite**”, “**Probable**”, and “**Possible**”. For the purposes of the PREVAIL study, **Definite**, **Probable** and **Possible** were all considered positive. The categories of infection are as follows:

| Category 1 | **Deep surgical wound infection** |
| --- | --- |
| Category 2 | **Incisional (or superficial) surgical wound infection** |
| Category 3 | **Skin and soft‐tissue infection (non‐surgical)** |
| Category 4 | **Catheter‐related blood stream infections** |
| Category 5 | **Primary blood stream infections** |
| Category 6 | **Lower urinary tract infection** |
| Category 7 | **Upper urinary tract infection** |
| Category 8 | **Intra‐abdominal infection** |
| Category 9 | **Sinusitis** |
| Category 10 | **Lower respiratory tract infection (excluding pneumonia)** |
| Category 11 | **ICU Acquired Pneumonia** |
| Category 12 | **Other** |

**CATEGORY 1: Deep surgical wound infection**

Deep surgical wound infection must meet the following criterion: Infection occurs at operative site within 30 days after surgery if no implant is left in place or within 1 year if implant is in place **AND** infection appears related to surgery **AND** infection involves tissues or spaces at or beneath fascial layer or a deeper anatomical space opened during the surgical procedure. In all categories, signs and symptoms suggestive of surgical site infection must be present. These include wound erythema and blanching, tenderness, pain, purulent discharge, fever, and leukocytosis.

**a) Definite Infection**An abscess or other evidence of infection seen on direct examination, during surgery or by histopathologic examination.

**OR**

Organism isolated from culture of fluid obtained during open procedure or aspiration

**b) Probable Infection**
Purulent drainage from drain placed beneath fascial layer (no microbial confirmation or Gram stain positive but negative culture).

**c) Possible Infection**
Wound spontaneously dehisces or is deliberately opened by surgeon (no pus or microbial confirmation).

**CATEGORY 2: Incisional (or superficial) surgical wound infection**

Incisional (or superficial) surgical wound infection must meet the following criterion: Infection occurs at incision site within 30 days after surgery **AND** involves skin and subcutaneous tissue above the fascial layer. In all categories, signs and symptoms suggestive of surgical site infection must be present. These include wound erythema and blanching, tenderness, pain, purulent discharge, fever, and leukocytosis.

**a) Definite Infection**
Organism(s) isolated from culture of fluid from wound closed primarily

**b) Probable Infection**
Purulent drainage from incision or drain located above fascial layer (no microbial confirmation or Gram stain positive but no positive culture).

**c) Possible Infection**
Surgeon deliberately opens wound because the clinical presence of infection

**CATEGORY 3: Skin and soft tissue infection (non‐surgical)**

Skin and soft tissue infections (non‐surgical) must meet the following criterion: Infection occurs in skin or soft tissue structures (SSTS) NOT associated with surgical procedures.

**a) Definite Infection**
Compelling clinical and laboratory evidence (such as spreading cutaneous erythema and blanching, or drainage or purulent material, with or without lymphangitis, in association with fever and leukocytosis) of the presence of SSTS infection based on clinical, radiographic, or surgical findings **AND** organism isolated from culture from a skin lesion that has drained pus or from a skin aspirate or biopsy of subcutaneous tissues of an erythematous skin lesion (not a simple skin swab).

**b) Probable Infection**
Compelling clinical and laboratory evidence (such as spreading cutaneous erythema and blanching, or drainage or purulent material, with or without lymphangitis, in association with fever and leukocytosis) or the presence of SSTS infection based on clinical, radiographic, or surgical findings **AND** no microbial confirmation or only positive Gram stain but negative culture.

**c) Possible Infection**
Some clinical evidence of infection, such as mild cutaneous erythema associated with fever, some laboratory evidence (leukocytosis), some radiographic but insufficient evidence to confirm a diagnosis **AND** no microbial confirmation

**CATEGORY 4: Catheter‐related blood stream infections (CRI)**

Catheter‐related blood stream infections (CRI) must be associated with an indwelling central line/arterial line (usually placed more than 5‐7 days ago) and have an organism isolated from the bloodstream that is not related to infection as some other site (lungs, GI tract, etc.). In addition, patients must have signs of sepsis (fever, chills, hypotension, etc.):

**a) Definite Catheter‐related Infection**
1. In association with a central line or arterial line, recognized pathogen (defined as a pathogen not usually regarded as a skin contaminant) isolated from one or more blood culture **AND** catheter tip positive (>15 CFU/mL) or hub or exit site culture positive with the same organism

**OR**

2. In association with a central line or arterial line, a common skin contaminant^[[2]](#footnote-2)^ isolated from two or more blood cultures (at least one from a venipuncture) **AND** catheter tip positive (>15 CFU/mL) or hub or exit site culture positive with the same organism

**b) Probable Infection**
1. In association with a central line or arterial line, recognized pathogen (defined as a pathogen not usually regarded as a skin contaminant) isolated from one or more blood culture

**OR**

2. In association with a central line or arterial line, a common skin contaminant isolated from two or more blood cultures (at least one from a venipuncture)

**c) Possible Infection**One of the following: fever (core temp >38°C), chills, or hypotension in association with a central line or arterial line (with or without a positive catheter tip (>15 CFU/ml) or positive hub or exit site positive)^[[3]](#footnote-3)^ **AND** patient’s clinical course improves with removal or change of the central line or arterial line and institution of appropriate antibiotic therapy.

**CATEGORY 5: Primary blood stream infections (BSI)**

Primary blood stream infections (BSI) must NOT be associated with an indwelling vascular device or related to infection as some other site (lungs, GI tract, etc.). In addition, patients must have signs of sepsis (fever, chills, hypotension, etc.):

**a) Definite Blood Stream Infection**
1. A recognized pathogen (defined as a pathogen not usually regarded as a skin contaminant) isolated from one or more blood culture

**OR**

2. A common skin contaminant^[[4]](#footnote-4)^ isolated from two or more blood cultures drawn on separate occasions (from venipunctures; must not be associated with a indwelling vascular device)

**c) Possible Infection**
A common skin contaminant isolated from a blood culture that does not fulfill the definition of ‘Definite” BSI **AND** patients’ clinical course improves with institution of appropriate antibiotic therapy.

**CATEGORY 6: Lower urinary tract infection (LUTI)**

**a) Definite**Symptoms (fever ‐ core temp > 38°C), hypotension) and Pyuria (> 10 white blood cells {WBC}/ml **AND** a positive urine culture of >10^5^ colonies/ml urine with no more than two species of organisms **AND** no other sources of the patient’s signs and symptoms are identified

**b) Probable**Symptoms (fever ‐ core temp > 38°C), hypotension) **AND** a urine culture of >10^5^ colonies/ml urine with no more than two species of organisms

**c) Possible^[[5]](#footnote-5)^**
A urine culture of >10^5^ colonies/ml urine with no more than two species of organisms

**CATEGORY 7: Upper Urinary Tract Infection**

Upper Urinary Tract Infection includes infections of the urinary tract (kidney, ureter, bladder, urethra, or perinephric spaces).

**a) Definite:**
Organism isolated from culture of fluid (other than urine) or tissue from affected site

**OR**

An abscess or other evidence of infection seen on direct examination, during surgery, or by histopathologic examination.

**b) Probable**Two of the following: fever (core temp >38°C), urgency, localized pain, or tenderness at involved site **AND** any of the following:

(a) Purulent drainage from affected site
(b) Positive Gram stain from fluid from affected site
(c) Organism isolated from urine or blood culture
(d) Radiographic evidence of infection

**c) Possible**Two of the following: fever (core temp >38°C), localized pain, or tenderness at involved site **AND**

1. Physician’s diagnosis
2. Physician institutes appropriate antimicrobial therapy and patient responds appropriately.

**CATEGORY 8: Intra‐abdominal infection**

Intra‐abdominal infection includes gallbladder, bile ducts, liver [other than viral hepatitis], spleen, pancreas, peritoneum, subphrenic or subdiaphragmatic space, pelvis or other intra‐abdominal tissue or area not specified elsewhere, and must meet the following criteria:

**a) Definite**
1. Organism(s) isolated from culture of purulent material from intra abdominal space/structure obtained during surgery or needle aspiration.

**OR**

2. Abscess or other evidence of intra‐abdominal infection (such as soilage of the peritoneal cavity after intestinal perforation) seen during surgery or by histopathologic examination.

**OR**

3. Pseudomembranous colitis‐ Direct visualization of pseudomembranes during sigmoidoscopy or on examination of surgically removed specimens of the colon.

**b) Probable**1. In the appropriate clinical setting, **organism isolated from blood culture** and radiographic evidence for intra‐abdominal infection

**OR**

Clinical evidence of intra‐abdominal infection (Abdominal Pain, Systemic leukocytosis, tenderness, jaundice)

**OR**

Laboratory evidence of intra‐abdominal infection (inflammatory ascitic fluid i.e. > 500 PMN/ml, evidence of biliary obstruction, positive gram stain of fluid from abdominal cavity but negative cultures).

2. Organisms seen on Gram stain of drainage or tissue obtained during surgery or needle aspiration but cultures are negative.

3. Pseudomembranous colitis‐ Toxin isolated from the stool in the setting of clinical illness compatible with Pseudomembranous colitis (exposure to antibiotics, diarrhea, colonic dilation, toxic megacolon, etc.)

**c) Possible:** one of the following:
1. Upper gastro‐intestinal perforation or penetrating abdominal trauma that is surgically repaired without further evidence of microbiologic confirmation or clinical signs or symptoms supportive of a diagnosis of bacterial or fungal peritonitis

2. Clinical evidence of intra‐abdominal infection with an inflammatory peritoneal fluid (> 500 leucocytes/ml for primary peritonitis and >100 leukocytes/ml for peritoneal dialysis related peritonitis) in the absence of a positive culture (in peritoneal fluid or blood) or gram stain

3. Organism isolated from culture of drainage from surgically placed drain (e.g., closed suction drainage system, open drain or T‐tube drain).

4. Clinical evidence of intra‐abdominal infection with persistent signs of systemic inflammation but without clear documented evidence of persistent inflammation within the peritoneal space following secondary bacterial peritonitis.

5. Clinical evidence of intra‐abdominal infection with signs of systemic inflammation which improves with the institution of systemic antibiotics (e.g. cholecystitis treated with antibiotics only)

6. Pseudomembranous colitis‐ Pseudomembranous colitis suspected on clinical grounds but toxin not sent or negative, colonoscopy not done, and therapy instituted.

**CATEGORY 9: Sinusitis**

**a) Definite**
Organism isolated from culture of purulent material directly obtained from sinus cavity by antral puncture.

**b) Probable**
One of the following: fever (core temp >38°C), pain or tenderness over the involved sinus, headache, purulent exudate, or nasal obstruction **AND** radiographic evidence of infection

**c) Possible**
Two of the following: fever (core temp >38°C) or pain or tenderness over the involved sinus, headache **AND** purulent nasal exudate.

**CATEGORY 10: Lower respiratory tract infection (excluding pneumonia)**

Lower respiratory tract infection (excluding pneumonia) includes infections such as bronchitis, tracheobronchitis, bronchiolitis, tracheitis, lung abscess, and empyema.

**a) Definite:**
Organism seen on smear or isolated from culture of lung tissue or fluid, including pleural fluid.

**b) Probable:**
1. Lung abscess or empyema seen during surgery or by histopathologic examination but no microbiological confirmation.
2. For bronchitis, tracheobronchitis, bronchiolitis, tracheitis, without evidence of pneumonia, must meet the following criterion: Patient has no clinical or radiographic evidence of pneumonia but has fever (core temp >38°C) and increased sputum production **AND** organism isolated from culture obtained by deep tracheal aspirate or bronchoscopy.

**c) Possible**
Abscess cavity seen on radiographic examination of lung.

**CATEGORY 11: ICU‐Acquired Pneumonia**

ICU‐Acquired Pneumonia includes HAP and VAP. It must be associated with a clinical suspicion of pneumonia defined as new, progressive, or persistent infiltrates on CXR and be associated with signs and symptoms of infection (fever, leukocytosis, worsening oxygenation, purulent secretions, etc.).

**a) Definite Pneumonia**
1. Radiographic evidence of pulmonary abscess and positive needle aspirate

**OR**

2. Histological proof on open lung biopsy or at post mortem (abscess formation, or consolidation with

PMN accumulation).

**b) Probable Pneumonia**
Must be associated with a positive culture of a pathogen known to cause pneumonia. For example, positive cultures for Coagulase Negative Staph. Species or normal oral flora would not be considered as positive since they do not usually cause VAP/HAP. The positive cultures need to come from 1 of the following:

1. A sputum or an endotracheal aspirate specimen.
2. A culture of bronchial washings, BAL or PSB regardless of quantitation (if done).
3. A blood culture of an organism found within 48 hours of the clinical suspicion of VAP/HAP.
4. A positive pleural fluid culture.

**c) Possible Pneumonia**
No microbial confirmation in the setting of a clinical suspicion for pneumonia as described above, and a clinical course compatible with VAP/HAP including the institution of appropriate antimicrobial therapy.

**CATEGORY 12: Other**

An infection that does not fall into any of the previous categories.

**a) Definite**
Clinical evidence of infection and one of the following: The culture of an organism(s) or positive Gram stain or positive viral cultures from a normally sterile bodily fluid or tissue in the absence of previous surgical intervention (e.g. organism isolated from CSF or synovial fluid).

**OR**

Positive antigen/RNA/DNA test for pathogens from a normally sterile bodily fluid

**OR**

Positive viral/bacterial serology.

**b) Probable**Clinical evidence of infection and of one the following: The culture of a pathogenic organism(s) or positive Gram stain positive or positive viral culture from a body site that is not normally sterile or a specimen obtained from an indwelling drain or catheter placed into a normally sterile body site (e.g. intra‐abdominal drain)

**OR**

Positive antigen/RNA/DNA test for pathogens from a body site that is not normally sterile.

**c) Possible**
Clinical evidence of infection but no microbiologic, smear or serological confirmation of infection

**Supplementary Digital Content 2: Multivariate analysis**

Table 1: Peak TNF-α logistic regression model

|  | **Parameter** | **Odds Ratio Estimate** | **Upper 95% Confidence Limit** | **Lower 95% Confidence Limit** | **P-Value** |
| --- | --- | --- | --- | --- | --- |
|  | **Low Peak TNF-α Tertile** | 0.67 | 0.30 | 1.52 | 0.32 |
|  | **Medium Peak TNF-α Tertile** | 0.94 | 0.42 | 2.07 | 0.71 |
|  | **Lactoferrin vs Placebo arm** | 0.85 | 0.44 | 1.67 | 0.64 |
|  | **Age** | 0.98 | 0.96 | 1.01 | 0.17 |
|  | **Female Sex** | 1.60 | 0.81 | 3.17 | 0.18 |
|  | **Admission Type Medical** | 0.47 | 0.20 | 1.10 | 0.08 |
|  | **APACHE II** | 0.97 | 0.92 | 1.02 | 0.18 |
|  | **Severe Sepsis** | 0.74 | 0.36 | 1.49 | 0.39 |

Table 2: Delta TNF-α logistic regression model

|  | **Parameter** | **Odds Ratio Estimate** | **Upper 95% Confidence Limit** | **Lower 95% Confidence Limit** | **P-Value** |
| --- | --- | --- | --- | --- | --- |
|  | **Low Delta TNF-α Tertile** | 1.01 | 0.45 | 2.30 | 0.53 |
|  | **Medium Delta TNF-α Tertile** | 0.65 | 0.29 | 1.46 | 0.23 |
|  | **Lactoferrin vs Placebo arm** | 0.81 | 0.41 | 1.59 | 0.54 |
|  | **Age** | 0.98 | 0.96 | 1.01 | 0.13 |
|  | **Female Sex** | 1.62 | 0.81 | 3.21 | 0.17 |
|  | **Admission Type Medical** | 0.45 | 0.19 | 1.06 | 0.07 |
|  | **APACHE II** | 0.97 | 0.93 | 1.02 | 0.22 |
|  | **Severe Sepsis** | 0.75 | 0.37 | 1.52 | 0.42 |

1. Calandra, T., J. Cohen, and I.S.F.D.o.I.i.t.I.C. Conference, *The international sepsis forum consensus conference on definitions of infection in the intensive care unit.* Crit Care Med, 2005. **33**(7): p. 1538-48. [↑](#footnote-ref-1)
2. Skin contaminants include diptheroids, Bacillus species, Propionibacterium, coagulase‐negative Staphylococci, or micrococci) [↑](#footnote-ref-2)
3. A positive catheter tip culture (>15 CFU/mL) or positive exit site culture without systemic symptoms and improvement with removal or change of the central/arterial line and institution of appropriate antibiotic therapy is not considered to be indicative of a central/arterial line infection [↑](#footnote-ref-3)
4. Skin contaminants include diptheroids, Bacillus species, Propionibacterium, coagulase‐negative Staphylococci, or micrococci) [↑](#footnote-ref-4)
5. Candida isolated in the urine may be considered indicative of a possible UTI if the attending physician feels that it is significant and institutes management for it (either/both changes the catheter or institutes antifungal therapy) [↑](#footnote-ref-5)
